# Colonoscopy-based *Clostridioides difficile* screening for inflammatory bowel disease patients

**DOI:** 10.1080/19490976.2026.2756799

**Published:** 2026-11-08

**Authors:** Jessica R. Allegretti, Alexander Carlin, Emma McClure, Mary Delaney, Lynn Bry

**Affiliations:** a Division of Gastroenterology, Hepatology and Endoscopy, Brigham and Women’s Hospital, Harvard Medical School, Boston, MA, USA; b Massachusetts Host-Microbiome Center, Department of Pathology, Brigham and Women’s Hospital, Harvard Medical School, Boston, MA, USA; c Clinical Microbiology Laboratory, Department of Pathology, Brigham and Women’s Hospital, Harvard Medical School, Boston, MA, USA

**Keywords:** Crohn’s disease, ulcerative colitis, inflammatory bowel disease, *Clostridioides difficile*

## Abstract

Patients with IBD have an increased risk for *Clostridioides difficile* infection, and the significance of asymptomatic colonization is poorly understood. Overlapping symptoms often complicate diagnosis, and there is a lack of validated screening methods. Colonic wash aspirates collected during routine colonoscopies may offer a convenient screening option. We aimed to evaluate culture methods for detecting *C. difficile* colonization in IBD patients using colonic wash samples. Patients with Crohn’s disease or ulcerative colitis undergoing outpatient colonoscopy were enrolled. Clinical characteristics, IBD activity indices, and endoscopic scores were recorded. Colonic wash aspirates were centrifuged and cultured on selective CHROMID® media with and without broth enrichment. Isolates were confirmed by Gram stain and MALDI-TOF. PCR/qPCR identified *tcdB* gene carriage. Whole-genome sequencing was performed on selected isolates, and associations between clinical factors and colonization were assessed. Of the 121 patients enrolled, 10.7% (13/121) were colonized. Crohn’s patients were colonized more frequently than ulcerative colitis patients (13.3% CD, 8.2% UC). Among colonized patients, 53.8% (7/13) had *C. difficile* with detectable *tcdB*. Enrichment increased detection compared to direct plating. No clinical predictors of colonization were identified, but wash samples yielded sufficient DNA for sequencing with comparable depth to whole stool. Colonoscopy wash aspirates allow for the detection of asymptomatic *C. difficile* colonization in IBD. This screening method may provide a useful tool for investigating the prevalence, persistence, and clinical significance of *C. difficile* colonization in IBD and for informing future studies of CDI prevention.

## Background

*Clostridioides difficile* is an obligate, sporulating anaerobe that can cause *C. difficile* infection (CDI), with clinical manifestations ranging from mild diarrhea to severe pseudomembranous colitis.^1^ Toxigenic strains of *C. difficile* cause diseases via their carriage of host-damaging toxins, including the *tcdA* and *tcdB* toxins, as well as the binary *cdtAB* toxin, which is found in many epidemic strains.^2^ CDI is the most common cause of nosocomial gastroenteritis in the United States, for which chronic exposure to the healthcare system increases risks for colonization with toxigenic strains and for the development of CDI.^3^

Patients with inflammatory bowel disease (IBD) are at increased risk for CDI. Diagnosis remains challenging as symptoms for both disease states are very similar, and *C. difficile* colonization is common in this population.^4^ Early identification and treatment of CDI are critical, which is why all IBD patients presenting with new symptoms should be tested for CDI.^5^ Asymptomatic carriage has been associated with subsequent CDI in other patient populations; however, the significance of *C. difficile* colonization among asymptomatic IBD patients is poorly understood.^3^ Identifying colonized patients is therefore an important first step towards understanding the clinical implications of carriage and evaluating potential preventive strategies such as decolonization. Currently, formed stool in an asymptomatic patient cannot be tested to assess for colonization. Therefore, the ability to identify *C. difficile* from colonic washes collected during a colonoscopy would streamline this testing; however, the feasibility of this approach has been debated, as these samples are considered to be diluted. We therefore aimed to develop a culture-based screening method for detecting asymptomatic *C. difficile* carriage from colonic wash samples collected during routine colonoscopy in patients with IBD. Secondary objectives were to evaluate the prevalence of carriage, clinical associations, and feasibility of genomic characterization of recovered isolates.

## Methods

Crohn’s disease (CD) or ulcerative colitis (UC) patients undergoing an outpatient colonoscopy for any indication were enrolled. Patients were considered asymptomatic with respect to CDI if they presented without clinical concern for active CDI and did not have new or unexplained diarrhea or other symptoms prompting CDI evaluation at the time of enrollment. Clinical characteristics, including IBD history, medication use history, history of prior *C. difficile* infection, and IBD clinical scores, including Harvy Bradshaw Index and Partial Mayo Score were collected. During the colonoscopy, IBD endoscopic scores were also assessed. A minimum of 20 mL of fluid was aspirated immediately upon entry into the colon, prior to water or air insufflation.

### Culture methods for C. difficile

Colonoscopy wash samples were received and stored at −80 °C until processing. Samples were thawed at room temperature, and 10 mL was centrifuged at 5000g for 5 minutes. 9 mL of the supernatant was decanted, and the pellet was resuspended in the remaining supernatant.

For direct culture, concentrated colonic wash pellets were plated directly onto CHROMID® *C. difficile* agar (bioMérieux, Marcy-l'Étoile, France) following resuspension, without prior broth enrichment. For culture-based enrichment of wash pellets, the centrifuged materials were added to 4 mL of pre-reduced BHI Broth with 0.1% taurocholate, D-cycloserine (125 mg/L), and cefoxitin (4 mg/L) and incubated for 48 hours. Thereafter, materials were plated to CHROMID® plates. Plates and enhanced BHI broths were incubated anaerobically at 37 °C for 48 hours in a Coy anaerobic chamber (Coy Laboratory Products, Inc., Grass Lake, Michigan) using AnO2 gas mix (10% H2, 10% CO2, and 80% N2) from MediTech gas. Plates were evaluated for *C. difficile* growth by the presence of black colonies with irregular edges at 24 and 48 hours. Isolates were Gram stained and subjected to MALDI-TOF identification (bioMérieux Inc., Durham, NC). For isolates lacking definitive typing by MALDI-TOF, defined as a Vitek MALDI ID < 80%, identification was reflexed into the RapID™ ANA panel (Remel) for species confirmation. The workflow for processing colonoscopy wash samples can be seen in Figure 1.

**Figure 1.**
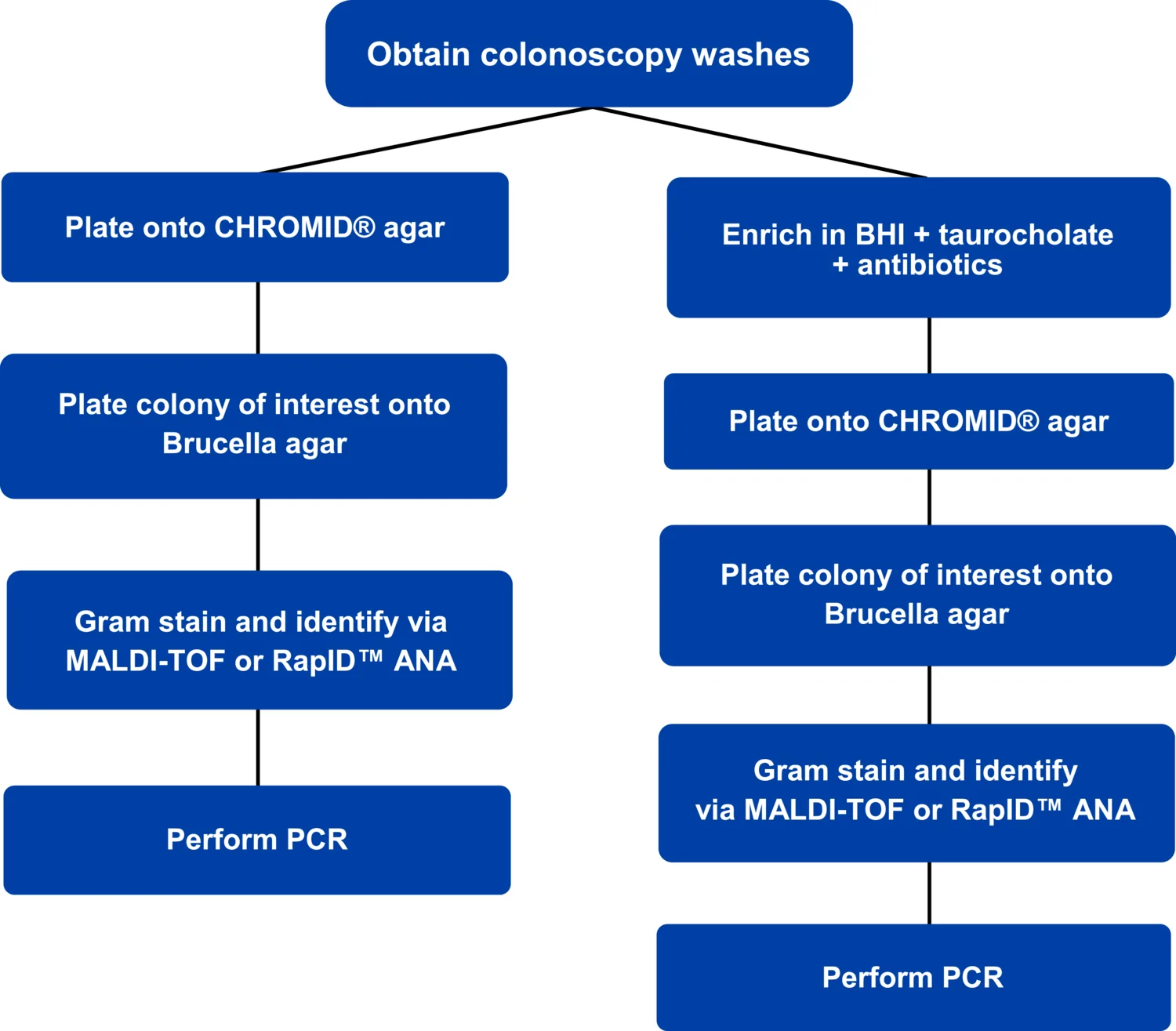
Workflow for *C. difficile* screening of colonoscopy washes. Colonoscopy washes were thawed, centrifuged, decanted, and resuspended prior to plating onto CHROMID® agar and BHI enrichment. Upon CHROMID® plating, colonies with *C. difficile* morphology were plated onto Brucella agar. Isolates were then Gram stained, and Gram-positive rods were identified via mass spectrometry (MALDI-TOF) or RapID™ ANA. *C. difficile* isolates were subjected to qPCR or endpoint PCR to determine tcdB carriage.

### C. difficile toxin carriage

DNA extraction was performed using the Zymo Quick-DNA Fecal/Soil Microbe Miniprep Kit (Zymo Research, Irvine, California). Endpoint PCR or qPCR was subsequently used to determine the carriage of the *tcdB* (toxin B) gene specifically. PCR products underwent gel electrophoresis using the PowerPac™ Basic (Bio-Rad Laboratories, Hercules, California) and Owl D3 gel electrophoresis system (Owl Separation Systems, Inc., Portsmouth, New Hampshire). PCR products were run on a 1% agarose gel at 100 volts for 25 minutes, then visualized under UV light using the 816 A UV light box (Fisher Scientific, Hampton, New Hampshire). For qPCR, eluted DNA was run in triplicate using TaqMan™ Multiplex Master Mix (Applied Biosystems) and the primers and probes used for endpoint PCR. The QuantStudio 12 K Flex instrument (Applied Biosystems), with a 40-cycle amplification at 95.0 °C (15 seconds per cycle) and 60.0 °C (1 minute), was used to determine the presence/absence of the *tcdB* gene.

### Whole genome sequencing

DNA from samples was isolated using the QIAGEN DNeasy PowerSoil Pro Kit, according to the manufacturer’s protocol. DNA samples were quantified using the GloMax Plate Reader System (Promega) using the QuantiFluor® dsDNA System (Promega) chemistry. DNA libraries were prepared using the Nextera XT DNA Library Preparation Kit (Illumina) and IDT Unique Dual Indexes with a total DNA input of 1 ng. Genomic DNA was fragmented using a proportional amount of Illumina Nextera XT fragmentation enzyme. Unique dual indexes were added to each sample, followed by 12 cycles of PCR to construct libraries. DNA libraries were purified using AMpure magnetic Beads (Beckman Coulter) and eluted in QIAGEN EB buffer. DNA libraries were quantified using Qubit 4 fluorometer and Qubit dsDNA HS Assay Kit. Libraries were then sequenced on the Illumina NovaSeq S4 platform 2 × 150 bp.

### Statistical analysis

A cross-tabulation analysis was performed with the two-tailed Fisher exact test on all categorical data to investigate each variable’s relationship with *C. difficile* colonization, including comparisons involving more than two categories. Patient age and *C. difficile* colonization were analyzed using a two-tailed Welch’s t-test, which does not assume equal variances. Age distributions were examined for departures from normality, and a Mann-Whitney U test was performed as a sensitivity analysis. Univariable logistic regression analyzes were performed to evaluate the relationship between individual clinical characteristics and *C. difficile* colonization, with colonization status as the binary outcome. Variables evaluated included IBD subtype, sex, prior surgery, biologic therapy, other IBD medications, corticosteroid therapy, and active disease. Given the small number of colonized patients, these analyzes were considered exploratory.

### Bioinformatics analysis

Sequencing data were analyzed using the CosmosID microbial genomics platform (CosmosID, Inc., Gaithersburg, MD, U.S.A.). The platform uses a curated database of publicly available microbial genomes and virulence markers. During database preparation, reference genomes are subdivided into variable-length sequence signatures (k-mers), which are categorized as shared or unique biomarkers and organized according to phylogenetic relationships. For each sample, sequencing reads and assembled contigs are queried against this biomarker database. Taxonomic classification is based on matching k-mers and reference biomarkers, with taxon abundance estimated from biomarker matching, sequence coverage, and genome size. Virulence-associated genes are identified by searching assembled sequences against the curated virulence-marker database. Reference genomes were obtained from NCBI GenBank and curated by CosmosID.

## Results

Between June 2020 and June 2021, a total of 121 patients were enrolled (60 CD and 61 UC). Demographic and clinical characteristics of the study population are summarized in Table 1. Among the UC cohort, 67% were female, mean age 42 ± 16, 66% had pancolitis, and 63.9% were on a biologic. The mean Mayo endoscopic score was 0.62; however, 60.7% had a score of 0. Among the CD cohort, 65% were female, mean age 37 ± 17, 50% had ileocolonic disease, and 75% were on a biologic. The mean SES-CD score was 2.4; however, 66.7% had a score of 0. Overall, 10.7% (13/121) of patients were found to be colonized with a *C. difficile* isolate at the time of colonoscopy, 13.3% (8/60) of CD patients, and 8.2% (5/61) of UC patients. Of those found to be colonized, 53.8% (7/13) had detectable *tcdB* positive *C. difficile*. A subgroup of nine samples underwent GDH testing. Notably, GDH-positive and *tcdB* toxin-negative *C. difficile* strains were isolated from the colonic wash samples of 2 CD and 2 UC patients. As described in Table 2, no statistically significant relationships were found between patient clinical characteristics and *C. difficile* colonization. Additionally, univariable logistic regression analyzes demonstrated no predictive variables for the colonization status of study participants.

**Table 1.** Patient characteristics.

Characteristic	Ulcerative colitis (*n* = 61)	Crohn's disease (*n* = 60)
Demographics		
Female, *n* (%)	41 (67.2)	39 (65.0)
Age, y, mean ± SD	42 ± 16	37 ± 17
Age at Diagnosis, *n* (%)		
≤16	10 (16.4)	19 (31.7)
17–40	37 (60.6)	33 (55.0)
≥41	14 (23.0)	8 (13.3)
Active Disease, *n* (%)	28 (45.9)	25 (41.7)
Endoscopic score* (Mayo-ES or SES-CD), *n* (%)		
Normal	37 (60.7)	40 (66.7)
Mild	14 (23.0)	12 (20.0)
Moderate	6 (9.8)	7 (11.7)
Severe	4 (6.6)	1 (1.7)
Therapies at time of collection, *n* (%)		
Biologic	39 (63.9)	45 (75.0)
anti-TNF		
Remicade	8 (13.1)	13 (21.7)
Humira	10 (16.4)	13 (21.7)
anti-Integrin		
Entyvio	11 (18.0)	10 (16.7)
anti-IL23		
Stelara	4 (6.6)	7 (11.7)
5-ASAs	17 (27.9)	2 (3.3)
MTX and Thiopurine	2 (3.3)	14 (23.3)
Steroid	11 (18.0)	10 (16.7)

^*^ Note: SES-CD was defined as 0–2 = normal, 3–6 = mild, 7–16 = moderate, and 17+ = severe. Mayo-ES was defined as 0 = normal, 1 = mild, 2 = moderate, and 3 = severe.

**Table 2.** Clinical characteristics and *C. difficile* carriage.

Variable	*C. difficile* negative (*n* = 108)	*C. difficile* positive (*n* = 13)	*p*-value
Diagnosis, n			0.395
Ulcerative Colitis	56	5	
Crohn's Disease	52	8	
Gender, n			0.539
Female	70	10	
Male	38	3	
Age, y, mean ± SD	40 ± 15	39 ± 15	0.918
Age at Diagnosis, n			0.850
≤16	25	4	
17–40	63	7	
≥41	20	2	
Disease State, n			0.385
Active	49	4	
Inactive	59	9	
Endoscopic score* (Mayo-ES or SES-CD), n			0.741
Normal	67	10	
Mild - Moderate	36	3	
Severe	5	0	
Therapies at time of collection, n			
Biologic	73	11	0.340
5-ASAs	17	2	1
MTX and Thiopurine	13	3	0.377
Steroid	19	2	1

Note: *SES-CD was defined as 0–2 = normal, 3–6 = mild, 7–16 = moderate, and ≥ 17 = severe. Mayo-ES was defined as 0 = normal, 1 = mild, 2 = moderate, and 3 = severe. Continuous variables were evaluated using a two-tailed t-test assuming unequal variances and categorical variables were evaluated using the Fisher exact test.

### Performance of culture-based concentration and enrichment techniques

Twenty-five *C. difficile* isolates were cultured from the 13 colonized patient samples. In eight of these thirteen samples, an isolate was retrieved from both direct culture and enhanced BHI culture.

Using FDA-approved and selective culture media for *C. difficile,* we evaluated the effectiveness of centrifugation to concentrate spores in wash samples, followed by direct plating to selective media, under anaerobic conditions, or BHI-broth enrichment with no selective additives, or with 0.1% taurocholate, D-cycloserine (125 mg/L), and cefoxitin (4 mg/L) to stimulate spore germination and selective growth over the commensal microbiota. Centrifugation with BHI selective broth culture provided improved detection of *C. difficile* in 68.0% of isolates (*n* = 17/25) compared to direct culture.

Additionally, a comparison of DNA quantity among the colonic wash samples compared to whole stool was performed. After extraction, there was, on average, more DNA in the stool samples (85 ng/uL) than in the colonic wash samples (37 ng/uL). However, library prep concentrations are similar (2.98 ng/uL and 2.72 ng/uL). From the colonic wash samples, ample biomass was present, allowing for similar data to be generated as compared to the stool with a mean read depth of 20 million sequences per sample. Thus, adequate read depth and DNA coverage were achieved compared to whole stool.

## Discussion

We present a screening method for detecting *C. difficile* carriage in IBD patients, utilizing samples that can be easily collected during a scheduled outpatient colonoscopy. It is known that IBD patients are at increased risk for the development of CDI and experience worse outcomes from infection. Clinical risk factors have been identified, but screening for carriage has not been previously assessed. While no significant clinical markers for *C. difficile* colonization were found in this study, the absence of associations with clinical characteristics examined does not exclude a role for host factors in asymptomatic carriage. Host responses in the epithelium and mucosa not captured by clinical or endoscopic measures may contribute to persistence without symptomatic disease and warrant further investigation. While this study was underpowered, the higher incidence of asymptomatic colonization in patients with Crohn’s disease presents an interesting avenue for further study. This study did not include a head-to-head comparison of colonoscopy wash samples with stool culture from the same patients, and therefore the sensitivity and specificity of this approach for detecting *C. difficile* colonization cannot be determined. Prospective studies comparing colonoscopy wash samples with stool-based methods are needed to further validate this approach. Additionally, toxigenic carriage was assessed by detection of *tcdB* only; *tcdA* and binary toxins were not evaluated, limiting the characterization of the toxin profile of the recovered isolates.

Asymptomatic carriage of tcdB-positive *C. difficile* raises questions regarding the host and microbial factors that permit *C. difficile* colonization without clinical disease. Colonization resistance, the surrounding microbiota, and mucosal host responses may influence whether *C. difficile* persists without progressing to symptomatic CDI in patients with IBD. The ability to recover viable isolates from colonoscopy washes may facilitate future longitudinal studies of these factors.

The results of this study demonstrate that *C. difficile* colonization can be detected in patients with IBD, even among patients in remission. Selective culture for *C. difficile,* including species identification and confirmation of *tcdB* carriage, can be successfully performed from colon aspirates collected during colonoscopy, even in the setting of bowel preparation. While selective enrichment improved recovery of *C. difficile*, the enrichment conditions may favor organisms capable of germination and growth under these specific conditions and therefore may not fully reflect the in vivo *C. difficile* population. This limitation does not alter the primary objective of establishing a method for recovery of viable *C. difficile* from colonic wash samples. Although routine screening for asymptomatic carriage is not currently standard clinical practice, this approach may provide a useful tool for better understanding the significance of *C. difficile* colonization in patients with IBD. Further study is needed to determine whether asymptomatic carriage is associated with subsequent CDI, IBD flares, or *C. difficile* transmission, and whether identification of colonization could ultimately inform preventive strategies.
